# Dietary Phosphorus, Its Sources, and Mortality in Adults on Haemodialysis: The DIET-HD Study

**DOI:** 10.3390/nu14194064

**Published:** 2022-09-30

**Authors:** Guobin Su, Valeria Saglimbene, Germaine Wong, Amélie Bernier-Jean, Juan Jesus Carrero, Patrizia Natale, Marinella Ruospo, Jorgen Hegbrant, Jonathan C. Craig, Giovanni F. M. Strippoli

**Affiliations:** 1 Department of Nephrology, Guangdong Provincial Hospital of Chinese Medicine, The Second Affiliated Hospital, Guangzhou University of Chinese Medicine, Guangzhou 510000, China; 2 Department of Medical Epidemiology and Biostatistics, Karolinska Institutet, 17177 Stockholm, Sweden; 3 Department of Global Public Health, Karolinska Institutet, 17177 Stockholm, Sweden; 4 European Renal Nutrition Working Group of the European Renal Association-European Dialysis Transplant Association (ERA-EDTA), 43126 Parma, Italy; 5 Faculty of Medicine and Health, Sydney School of Public Health, The University of Sydney, Edward Ford Building A27, Sydney, NSW 2006, Australia; 6 Department of Emergency and Organ Transplantation, University of Bari, Piazza Giulio Cesare, 70124 Bari, Italy; 7 Centre for Kidney Research, The Children’s Hospital at Westmead, Sydney, NSW 2145, Australia; 8 Division of Nephrology, CIUSSS du Nord-de-l’Île-de-Montréal, Faculty of Medicine, Université de Montréal, Montréal, QC 2900, Canada; 9 Nephrology, Dialysis and Transplantation Unit, Department of Medical and Surgical Sciences, University of Foggia, 71122 Foggia, Italy; 10 Division of Nephrology, Department of Clinical Sciences, Lund University, 22100 Lund, Sweden; 11 College of Medicine and Public Health, Flinders University, Adelaide, SA 5042, Australia

**Keywords:** phosphorus, plant-base, mortality, haemodialysis, cohort, DIET-HD

## Abstract

Dietary phosphorus restrictions are usually recommended for people on haemodialysis, although its impact on patient-relevant outcomes is uncertain. We aimed to evaluate the association between total phosphorus intake and its sources with mortality in haemodialysis. Phosphorus intake was ascertained within the DIET-HD study in 8110 adults on haemodialysis. Adjusted Cox regression analyses were conducted to evaluate the association between the total and source-specific phosphorus (plant-, animal-, or processed and other sources) with mortality. During a median 3.8 years of follow-up, there were 2953 deaths, 1160 cardiovascular-related. The median phosphorus intake was 1388 mg/day. Every standard deviation (SD) (896 mg/day) increase in total phosphorus was associated with higher all-cause mortality [hazard ratio (HR), 1.16; 95% confidence intervals (CI), 1.06–1.26] and cardiovascular mortality (HR, 1.18; 95% CI, 1.03–1.36). Every SD (17%) increase in the proportion of phosphorus from plant sources was associated with lower all-cause mortality (HR, 0.95; 95% CI, 0.90–0.99). Every SD (9%) increase in the proportion of phosphorus from the processed and other sources was associated with higher all-cause mortality (HR, 1.06; 95% CI, 1.02–1.10). A higher total phosphorus intake was associated with increased all-cause and cardiovascular death. This association is driven largely by the phosphorus intake from processed food. Plant based phosphorus was associated with lower all-cause mortality.

## 1. Introduction

Hyperphosphatemia is common in stage V chronic kidney disease (CKD), affecting more than 50% of patients treated with haemodialysis [1,2]. It leads to mineral and bone metabolism disorder [3,4] and is strongly associated with increased cardiovascular disease and mortality in this population [5,6].

Dietary phosphorus restrictions are usually recommended to avoid hyperphosphatemia and its complications in haemodialysis [7,8,9]. Clinical practice guidelines [8,9] recommend limiting the phosphorus intake by considering the different bioavailability of different food sources of phosphorus. (20 to 40% from plant, 40 to 60% from animal, and up to 100% from additives) [10]. However, these guidelines do not recommend any specific change in intake based upon sources. They are informed by expert opinion only, with very limited epidemiological data on the impact of dietary phosphorus and its sources on patient-relevant outcomes. The few observational studies investigating the association between total phosphorus intake and mortality have shown inconsistent results and did not explore the role of different food sources [11,12].

The aim of this study was to evaluate the association between total phosphorus intake and phosphorus food sources (plant, animal, processed and other sources) with all-cause and cardiovascular mortality in people treated with long-term haemodialysis.

## 2. Material and Methods

### 2.1. Study Design

This study is a sub-analysis of the DIET-HD study, a multinational prospective cohort study evaluating the association between diet and adverse outcomes in adults treated with long-term haemodialysis [13]. This study is reported according to the Strengthening the Reporting of Observational Studies in Epidemiology (STROBE) guidelines [14].

### 2.2. Study Population

Adults undergoing haemodialysis in Europe (Italy, France, Germany, Hungary, Portugal, Poland, Sweden, Spain, Turkey, and Romania) and South America (Argentina) were eligible. Patients were excluded if they had a scheduled kidney transplant within 6 months or a life expectancy of less than 6 months, if unable to complete a Food Frequency Questionnaire (FFQ). The study was conducted in accordance with the Declaration of Helsinki. Ethics approval was obtained from all relevant institutional ethics committees. All patients provided written informed consent.

### 2.3. Dietary Assessment of Phosphorus Intake

Dietary intake was estimated using the Global Allergy and Asthma European Network (GA2LEN) FFQ, which comprised 32 food groups, and was validated to assess the dietary intake across many European countries [15]. Standard food portion sizes were used to calculate daily food intake in grams per day [16]. Micro- and macronutrient intake were derived using the latest McCance and Widdowson’s Food Composition Tables [17].

Plant-based phosphorus was calculated as the amount of phosphorus contributed by plant foods including fruits, vegetables, legumes, whole grains, beans, seeds, and nuts. Animal phosphorus sources included fish, eggs, meats, and dairy foods. Other sources of phosphorus included predominantly processed foods including sauces, cakes, and drinks (Supplementary Table S1). The total amount of daily phosphorus intake in a food equalled the sum of plant, animal, and other sources of phosphorus. The proportion of phosphorus from plant, animal, processed, and other sources was defined as the phosphorus intake from each of the three sources divided by the total daily phosphorus intake.

### 2.4. Outcomes

The study outcome was mortality, both all-cause and cardiovascular-related. Cardiovascular-related mortality was defined as death attributed to atherosclerotic heart disease, acute myocardial infarction, cardiac arrhythmia, cardiac arrest, congestive cardiac failure, pericarditis, pulmonary oedema, valvular heart disease, or cardiomyopathy, or sudden death. The causes of death were obtained from death certificates, which were filled in and adjudicated by the participants’ treating clinicians. The causes of death were collected in a centralised database using the same identifier and recorded according to the United States coding for the population with kidney failure [5].

### 2.5. Statistical Analysis

Baseline variables were calculated as the median and interquartile range or mean and standard deviation for continuous variables, according to their distribution, and as frequencies and percentages for categorical variables. The date of the FFQ administration was used as the index date to calculate the time at risk to the date of death or censoring for recovery of kidney function, kidney transplantation, transfer to peritoneal dialysis, withdrawal from dialysis, transfer outside the network, being temporary out of the dialysis network, end of the follow-up period, or lost to follow-up for other reasons. For cardiovascular-related mortality, participants who died from non-cardiovascular causes were censored.

### 2.6. Missing Data Handling

Participant surveys were excluded from the analysis if responses were implausible (defined as a log-transformed total energy intake more than three standard deviations from the mean) or incomplete for more than 20%. In the included subset, most covariates had <5% missing. Data were assumed to be missing at random and 25 datasets were imputed using the multiple imputation chained equation (Supplementary Item S1). Data for mortality outcomes were complete.

### 2.7. Main Analysis

Restricted cubic splines were used to evaluate the linearity of the relationships between the total and source-specific phosphorus intake and outcomes with three knots and the 10th percentile of each exposure as reference. No deviance from linearity was observed. We assessed the association with mortality of total phosphorus intake and proportion of phosphorus from different sources, treated both as continuous and tertiles, in a univariable and multivariable random effects shared frailty Cox proportional hazard regression model to account for clustering effects within countries.

Results were presented as hazard ratios (HR) with the associated 95% confidence intervals (CI) using the lowest tertile as the reference category or for every SD increase.

To build the full multivariable model, we entered covariates with univariable associations with mortality (*p* < 0.25) into a base model (plus the a priori forced variables sex, physical activity, and energy intake pre-specified as clinically relevant). No collinearity was found between these covariates (defined by variance inflation factor of 1). The final model was determined by backwards elimination until only forced variables, variables with multivariable *p* < 0.05, or >10% change in estimates remained. All analyses were adjusted for age, sex, smoking (never vs. ever), physical activity (none vs. any), body mass index (<25, 25–30, ≥30 kg/m^2^), education (secondary level or above), history of cardiovascular disease, hypertension, diabetes, pulmonary disease, cancer, serum haemoglobin, serum albumin, serum calcium, statins, phosphate-binders (any or none), vascular access type (fistula versus graft/catheter), dialysis vintage, Kt/V, total energy intake (1000 kcal/day), daily potassium intake, and Mediterranean diet score. The analysis of phosphorus from different sources (proportion) was also adjusted for the total phosphorus intake (mg per day). The analysis of cardiovascular mortality was additionally adjusted for alcohol consumption and antihypertensive drugs. No deviation from the proportional hazards assumption was found in the Cox models by fitting log (time)-dependent covariates in the multivariable model.

### 2.8. Subgroup Analysis

We tested for possible effect modification for the association between the exposure (the total phosphorus intake and proportion of phosphorus intake from different sources) and mortality outcomes by pre-specified variables sex, age [<65, ≥65 years], presence/absence of cardiovascular disease comorbidity (composite of angina, myocardial infarction, congestive heart failure, atrial fibrillation, and stroke), or diabetes.

### 2.9. Sensitivity Analysis

To investigate the role of serum phosphorus in the association between dietary phosphorus and mortality, we additionally adjusted the regression models for its value. Since restricting phosphorus typically results in reduced protein intake, hypothetically leading to undernutrition, we additionally adjusted the regression models for protein intake in a sensitivity analysis. A stratified proportional sub-distribution hazard model was used to consider the potential relevance of competing events (kidney transplantation for the analysis of all-cause mortality; kidney transplantation and death from non-cardiovascular causes for the analysis of cardiovascular mortality).

All analyses were conducted using the Stata 15.0 and the Free Statistics analysis platform. A two-tailed *p* < 0.05 was used as an indication of statistical significance.

## 3. Results

Out of the 9757 adults in the DIET-HD study, 8110 (83%) had complete diet data and were included in our analyses (Figure S1).

### 3.1. Baseline Characteristics

**Figure 1:**
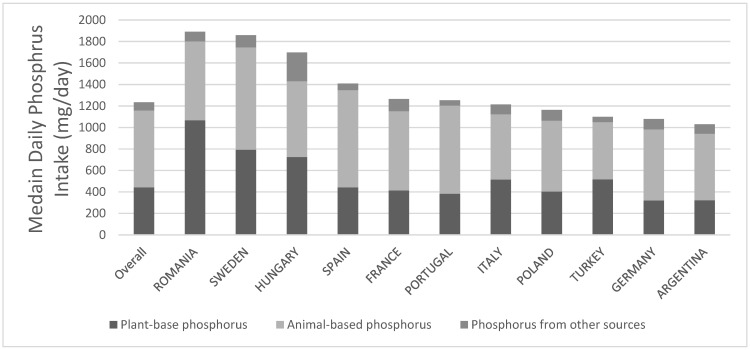
The median intake of dietary phosphorus (mg/day), stratified by food source and country.

The median phosphorus intake was 1388 (IQR 961-1994) mg/day. Overall, the major source of phosphorus was animal (57.9%), followed by plant (35.8%) and other sources including mostly processed food (6.3%). This pattern was broadly consistent across all countries (Figure 1). The baseline characteristics per tertile of phosphorus intake are summarised in Table 1. Patients with higher phosphorus intake were more likely to be male, to have cardiovascular disease, chronic pulmonary disease or cancer, to be less physically active, to consume alcohol, to have more energy and protein intake, to have higher serum phosphorus, and to receive phosphate-binders.

### 3.2. Total Phosphorus Intake and Mortality

During a median 3.8 years of follow-up (IQR, 1.7–4.7), there were 2953 deaths (36% of patients), of which 1160 (39%) were cardiovascular-related deaths.

**Figure 2:**
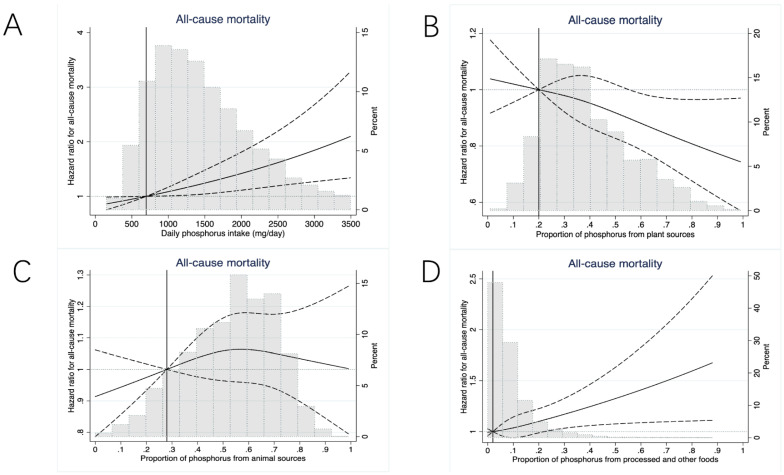
Adjusted restricted cubic splines for the association between phosphorus intake (**A**), proportion of phosphorus from plant (**B**), animal (**C**), processed and other sources (**D**), and all-cause mortality.

**Figure 3:**
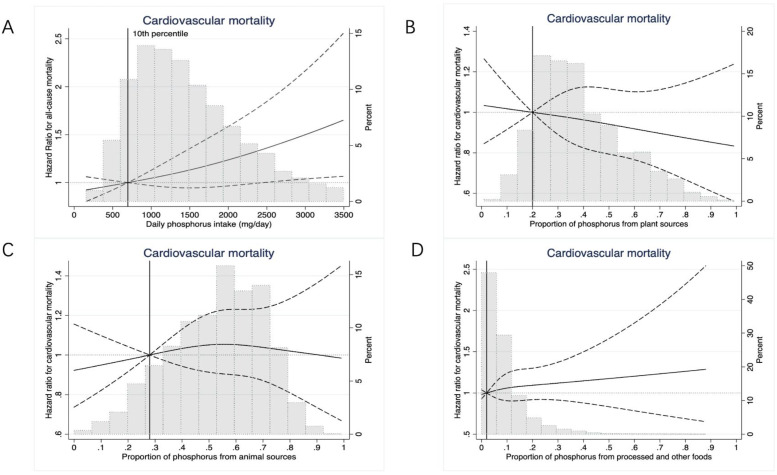
Adjusted restricted cubic splines of the association between daily phosphorus intake (**A**), proportion of phosphorus from plant (**B**), animal (**C**), other sources (**D**) and cardiovascular mortality.

**Figure 4:**
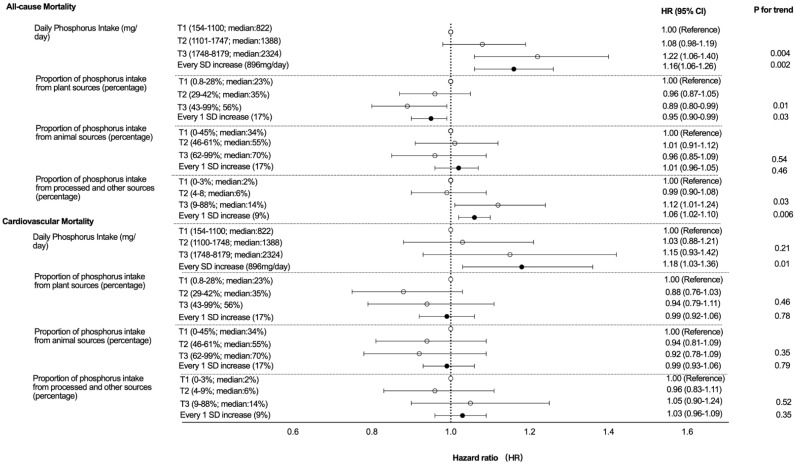
Forest plot of the adjusted association between the mortality and tertiles of total dietary phosphorus intake, and the proportion of phosphorus from plant, animal, and other sources.

Compared with patients in the lowest phosphorus intake tertile (154–1100 mg/day), the adjusted HRs for all-cause mortality among those in the middle (1101–1747 mg/day) and highest tertiles (1748–8179 mg/day) were 1.08 [95% confident interval (CI), 0.98–1.19] and 1.22 (95%CI, 1.06–1.26), respectively (*p* value per trend = 0.004). The adjusted HRs for cardiovascular mortality were respectively 1.09 [95% CI, 0.84–1.41] and 1.34 (95%CI, 0.91–1.98) (*p* value per trend = 0.21). Every standard deviation (SD, 896 mg/day) increase in phosphorus intake was associated with higher all-cause mortality [Hazard ratio (HR), 1.16; 95%CI, 1.06–1.26] and cardiovascular mortality (HR 1.18; 95%CI, 1.03–1.36) (Figure 2, Figure 3 and Figure 4). These results were consistent in the sensitivity analyses when adjusting for serum phosphorus, protein intake, and in competing risk analysis (Table S2).

Restricted cubic spline models adjusted for age, sex, physical activity, smoking, body mass index, secondary education, history of cardiovascular disease, hypertension, diabetes, pulmonary disease, cancer, serum haemoglobin, serum albumin, serum calcium, statins, phosphate-binders (any or none), vascular access type (fistula versus graft/catheter), dialysis vintage, Kt/V, total energy intake, potassium intake gram per day, and Mediterranean diet score (we included the total daily phosphorus intake as the covariate when investigating the association between the proportion of phosphorus from specific sources and mortality).

Restricted cubic spline models adjusted for age, sex, physical activity, smoking, body mass index, secondary education, history of cardiovascular disease, hypertension, diabetes, pulmonary disease, cancer, serum haemoglobin, serum albumin, serum calcium, statins, phosphate-binders (any or none), vascular access type (fistula versus graft/catheter), dialysis vintage, Kt/V, total energy intake per day, potassium intake gram per day, Mediterranean diet score and alcohol consumption, and antihypertensive drugs (we included the total daily phosphorus intake as the covariate when investigating the association between the proportion of phosphorus from specific sources and mortality).

Multivariable Cox proportional hazard regression analyses fitted using a random effects shared frailty model adjusted for age, sex, physical activity, smoking, body mass index, secondary education, history of cardiovascular disease, hypertension, diabetes, pulmonary disease, cancer, serum haemoglobin, serum albumin, serum calcium, statins, phosphate-binders (any or none), vascular access type (fistula versus graft/catheter), dialysis vintage, Kt/V, total energy intake per day and potassium intake gram per day, and Mediterranean diet score (we included the total daily phosphorus intake as the covariate when investigating the association between the proportion of phosphorus from specific sources and mortality). The analysis of cardiovascular mortality was additionally adjusted for alcohol consumption and antihypertensive drugs

### 3.3. Proportion of Phosphorus Intake from Different Sources and Mortality

#### 3.3.1. Plant Sources

Compared with patients in the lowest tertile of the proportion of phosphorus intake from plant sources (0.8–28%), the adjusted HRs for all-cause mortality among those in the middle (29–42%) and highest tertiles (43–99%) were 0.96 [95% CI, 0.87–1.05] and 0.89 (95%CI, 0.80–0.99), respectively (*p* value per trend = 0.01). The adjusted HRs for cardiovascular mortality were 0.88 [95% CI, 0.76–1.03] and 0.94 [95% CI, 0.79–1.11] for the middle and highest tertiles of phosphorus intake from the plant sources, respectively (*p* value per trend = 0.46). Every standard deviation (SD, 17%) increase in the proportion of phosphorus intake from plant sources was associated with lower all-cause mortality (HR 0.95; 95%CI: 0.90–0.99), but not with lower cardiovascular mortality (HR 0.99; 95%CI, 0.92–1.06) (Figure 2, Figure 3 and Figure 4).

#### 3.3.2. Animal Sources

Compared with patients in the lowest tertile of proportion of phosphorus intake from animal sources (0–45%), the adjusted HRs for all-cause mortality among those in the middle (46–61%) and highest tertiles (62–69%) were 1.01 [95% CI, 0.91–1.12] and 0.96 (95%CI, 0.85–1.09), respectively (*p* value per trend = 0.51). The adjusted HRs for cardiovascular mortality were 0.94 [95% CI, 0.81–1.09] and 0.92 [95% CI, 0.76–1.09] for the middle and highest tertiles of phosphorus intake from animal sources, respectively (*p* value per trend = 0.46). Every standard deviation (SD, 17%) increase in the proportion of phosphorus intake from animal sources was not associated with all-cause mortality (HR 1.01; 95%CI: 0.96–1.05) or cardiovascular mortality (HR 0.99; 95%CI, 0.93–1.06) (Figure 2, Figure 3 and Figure 4).

#### 3.3.3. Processed and Other Sources

Compared with patients in the lowest tertile of proportion of phosphorus intake from processed and other sources (0–3%), the adjusted HRs for all-cause mortality among those in the middle (4–8%) and highest (9–88%) tertile were 0.99 [95% CI, 0.90–1.08] and 1.12 (95%CI, 1.01–1.24), respectively (*p* value per trend = 0.03). The adjusted HRs for cardiovascular mortality were 0.96 [95% CI, 0.83–1.11] and 1.05 [95% CI, 0.90–1.24], respectively, for the middle and highest tertiles of phosphorus intake from the animal sources, respectively (*p* value per trend = 0.52). Every standard deviation (SD, 9%) increase in the proportion of phosphorus intake from other sources was associated with the increased all-cause mortality (HR 1.06; 95%CI: 1.02–1.10), but not with cardiovascular mortality (HR 1.03; 95%CI: 0.96–1.09) (Figure 2, Figure 3 and Figure 4).

The results were consistent with the sensitivity analyses when adjusting for serum phosphorus, protein intake, and in competing risk analysis (Tables S3–S5)

### 3.4. Subgroup Analysis

The association between exposure (total phosphorus intake and proportion of phosphorus intake from different sources) and mortality was not modified by sex, age [<65, ≥65 years], presence/absence of cardiovascular disease, or diabetes.

## 4. Discussion

In this multi-national prospective cohort study of people receiving long-term haemodialysis, with about four years of follow-up, we found that a higher total phosphorus intake was associated with both all-cause and cardiovascular mortality. For every daily increase of around 100 mg, there was an increase of 16% and 18% of all-cause and cardiovascular death, respectively. Investigating the impact of dietary phosphorus from different food sources, a higher proportion from plant sources was associated with reduced all-cause mortality. In contrast, a higher proportion of phosphorus intake from sources other than animal and plant, mostly processed food (such as sauces, cakes, and drinks) was associated with increased all-cause mortality. No association was found between dietary phosphorus intake from animal sources and all-cause mortality or cardiovascular mortality.

There is little previous evidence on the role of phosphorus intake on patient-relevant outcomes. Only two observational studies have evaluated the association between phosphorus intake and mortality in haemodialysis patients. These were single country studies, both conducted in the U.S., with smaller sample sizes, using different definitions of phosphorus intake and found discordant results. Neither investigated the role of different sources of phosphorus [11,12]. Our finding of a positive association between the total phosphorus intake and mortality is consistent with the results from one study [11] that assessed the phosphorus intake using a food frequency questionnaire. In contrast, the other assessed the prescribed phosphorus intake, instead of the measured phosphorus intake, without finding any association with mortality [12]. The prescribed lower phosphorus intake might have resulted in an unintentional reduced intake of other beneficial nutrients, hampering the possibility of observing a survival benefit of a lower phosphorus intake.

Many potential mechanisms might explain the increased mortality associated with the higher total phosphorus intake observed in our study. Higher phosphorus intake results in increased PTH, FGF23, and dopamine levels [3]. FGF23 has been associated with congestive heart failure, left ventricular hypertrophy, and mortality [18,19,20]. High phosphorus intake may also induce endothelial dysfunction, as shown by the impaired endothelium dependent vasodilatation observed in patients with stage 5 CKD when exposed to high concentrations of phosphorus [21].

In a crossover trial of patients with CKD G3, comparing the consumption of plant versus animal protein diet given the same amount of phosphorus content (800 mg per day), the plant diet resulted in an improved phosphate control and lower serum FGF-23 levels [22]. Similarly, in an uncontrolled 4-week study of patients with mild to advanced CKD, a plant-based diet was associated with lower urine phosphorus and better bicarbonate levels [23]. Whether a higher proportion of phosphorus intake from plants is associated with better survival through lower FGF23 or better bicarbonate levels needs further investigation. Given the results of a previous DIET-HD substudy that showed a survival benefit associated with higher fruit and vegetable intake (as food groups) in haemodialysis [24], we cannot exclude that other components of plant foods, together with phosphorus (such as flavonoids and/or fibre), might contribute to the survival benefit of phosphorus intake from plants observed in this analysis [25], although adjusted for the Mediterranean score, which takes into account food groups including fruits and vegetables.

The observed association between higher phosphorus intake from processed food and other sources and higher all-cause mortality might be driven by the inorganic phosphorus additives of the processed food considered in this analysis (sauces, cakes, and drinks). Indeed, inorganic phosphorus additives are highly absorbed by the digestive track (more than 90%), contributing significantly to hyperphosphatemia [26]. Therefore, higher phosphorus intake from processed food might result in hyperphosphatemia, which has been linked to higher infection-related mortality [27], contributing to increased all-cause mortality. In addition, the dietary intake of inorganic phosphate additives has been associated with poor bone health [28] and cancer related morbidities [29]. All of these might contribute to the observed increased all-cause mortality associated with higher phosphorus from processed and other sources.

Clinical practice guidelines for CKD mineral bone disease and nutrition recommend limiting dietary phosphate intake in the treatment of hyperphosphatemia by considering the different bioavailability of different food sources of phosphorus. However, these recommendations are not graded due to the lack of high certainty evidence and acknowledge the need for research on the role of phosphate quality (e.g., animal, vegetable, additives) [8,9]. Our study supports that, in addition to the total phosphorus intake, its sources also play an important role in the prognosis of haemodialysis patients by investigating their impact on patient-relevant outcomes. In particular, our findings suggest a survival benefit of controlling the total phosphorus intake by limiting the intake from processed and other sources in favour of the intake from plant sources. Intervention trials are required to confirm these findings and support the dietary recommendations for phosphorus intake in haemodialysis.

The strengths of our study include the prospective multinational design, which increases the generalisability of the findings. Furthermore, we expanded on previous studies by assessing the role of total phosphorus intake and its specific source on patient-relevant outcomes, providing supporting evidence to the currently not graded dietary recommendations on phosphorus intake in haemodialysis.

This study has several potential limitations. The phosphorus intake was self-reported and comprised a single measurement at baseline, which may have led to a measurement bias that is likely to drive the result towards null. The FFQ is validated for the general population, but not in haemodialysis, although it is not clear why the measurement properties would be different in this setting. We did not record the cooking methods or food additives [30] that might have influenced the actual dietary phosphorus intake. This may have underestimated the total dietary phosphorus intake, making our observations based primarily on organic phosphorus intake. However, this is compatible with how dietary phosphorus restrictions are advised in clinical practice. This was an observational study and not a randomised controlled trial. Therefore, there were inherent differences between the exposed and unexposed groups that may affect the internal and external validity of the study results. To address the issue of confounding bias, the potential confounders were included in the regression model as explanatory factors, and adjusted estimates were provided, although, as in any observational study, residual confounding could not be excluded. Selection bias was minimised by including consecutive patients at the participating sites, and missingness (missing at random) was handled using multiple imputation. Findings were confirmed in sensitivity analyses adjusting for additional potential confounders (such as serum phosphorus and protein intake) and by assessing the potential relevance of competing events. We were unable to assess the temporal change (and variability) in dietary phosphate intake and its association with mortality. This is a relevant research question because the dietary intake of an individual is unlikely to be constant over time, and there may be considerable between and within person variability for different food and nutrient intake. The association between dietary pattern changes over time and mortality should be investigated in future research. We acknowledge a mediation analysis using serum phosphorus as a mediator to assess the association between phosphorus intake and mortality using a counterfactual model, which would allow us to define the causal interpretation of the association. However, this would require the assessment of many other unmeasured (e.g., PTH and vitamin D) and unknown factors and the interactive effects with serum phosphorus (as the mediator) and phosphorus intake (as the exposure). These variables are currently not available.

In conclusion, a higher total phosphorus intake is associated with increased death. This positive association is likely to be driven by the phosphorus intake from sources that are neither animal nor plant such as processed food, while a higher proportion of phosphorus intake from plants is associated with reduced all-cause mortality. Intervention studies are needed to support the dietary recommendations on phosphorus intake from specific food sources in haemodialysis.

## Figures and Tables

**Table 1:** The baseline characteristics, overall, and by tertiles of the daily phosphorus intake.

Characteristics	Overall (*n* = 8110)	Lowest Tertile (154–1100 mg/Day) (*n* = 2704)	Middle Tertile (1101–1747 g/Day) (*n* = 2703)	Highest Tertile (1748–8179 g/day) (*n* = 2703)	*p* for Trend
**Demographics**					
Age, years, mean (SD)	63 (15)	63 (15)	64 (15)	62 (15)	0.77
Women, *n* (%)	3419 (42)	1209 (45)	1156 (43)	1054 (39)	<0.01
Education: Secondary level or above, *n* (%)	2699 (44)	1158 (43)	1253 (46)	1314 (49)	<0.01
Never smoker, *n* (%)	4212 (67)	1854 (69)	1807 (67)	1796 (66)	0.1
Irregular physical activity or more, *n* (%)	3249 (52)	1391 (51)	1369 (51)	1559 (58)	<0.01
Never drinking alcohol, *n* (%)	4116 (51)	1455 (54)	1376 (51)	1282 (47)	<0.01
Body mass index, *n* (%)	26 (5)	26(5)	26(5)	26 (6)	0.9
**Diet**					
Energy intake, kcal/d, median (IQR)	1900 (1404–2520)	1260 (992–1552)	1971 (1682–2286)	2762 (2228–3450)	<0.01
Potassium intake, g/d, median (IQR)	1388 (961–1994)	882 (654–961)	1389 (1243–1557)	2324(1995–2858)	<0.01
Mediterranean score, median (IQR)	4 (3–5)	3 (2–4)	4 (3–5)	5 (4–6)	<0.01
**Clinical characteristics**					
Weight change in kg (3 months prior), median (IQR)	0.1 (−0.9–1)	0.1 (−1–1)	0.0 (−0.9–1)	0 (−0.1–1)	0.45
Hypertension, *n* (%)	6219 (85)	2272 (84)	2323 (86)	2301 (85)	0.26
Diabetes mellitus, *n* (%)	2332 (32)	834 (31)	895 (33)	848 (31)	0.68
Cardiovascular disease, *n* (%)	2973 (37)	880 (33)	1033 (38)	1060 (39)	<0.01
Chronic pulmonary disease, *n* (%)	940 (12)	276 (10)	312 (12)	352 (13)	<0.01
Cancer, *n* (%)	1045 (13)	285 (10)	381 (14)	379 (14)	<0.01
**Laboratory variables**					
Normalised protein catabolic rate, g/kg/d, median (IQR)	1.1 (0.9–1.3)	1.1 (0.9–1.3)	1.1 (0.9–1.3)	1.1 (0.9–1.3)	0.45
Serum phosphorus, mg/dl, mean (SD)	4.7 (1.4)	4.7 (1.4)	4.6 (1.4)	4.8 (1.5)	<0.01
Haemoglobin, g/dl, mean (SD)	11.1 (1.3)	11.1 (1.3)	11.1 (1.3)	11.0 (1.3)	0.15
Albumin, g/l, mean (SD)	39.8 (3.8)	39.7 (3.8)	39.9 (3.7)	39.8 (3.8)	0.31
Calcium, mg/dl, mean (SD)	8.9 (0.7)	8.9 (0.7)	9.0 (0.7)	8.9 (0.7)	0.37
**Dialysis characteristics**					
Arteriovenous fistula, *n* (%)	6481 (81)	2157 (80)	2168 (80)	2208 (82)	0.08
Dialysis vintage, years, median (IQR)	3.6 (1.7–6.8)	3.8 (1.8–7.1)	3.6 (1.7–6.8)	3.5 (1.7–6.5)	0.19
Kt/V, mean (SD)	1.7 (0.3)	1.8 (0.4)	1.8 (0.3)	1.7 (0.3)	<0.01
**Medications**					
Antihypertensives, *n* (%)	6136 (76)	2039 (75)	2036 (75)	2061 (76)	0.47
Phosphate binders, *n* (%)	3263 (40)	895 (33)	1065 (39)	1303 (48)	<0.01
Statin, *n* (%)	2316 (37)	960 (36)	1025 (38)	961 (36)	0.96

Cardiovascular disease includes myocardial infarction, angina, atrial fibrillation, congestive heart failure, cardiac arrest, stroke. Antihypertensive drugs include angiotensin II receptor blocker, angiotensin-converting enzyme inhibitors, calcium antagonists, beta blockers, vasodilators, diuretics, and alfa block.

## Data Availability

The data presented in this study are available on request from the corresponding author.

## Supplementary Materials

The following are available online at https://www.mdpi.com/article/10.3390/nu14194064/s1, Figure S1. Flowchart; Table S1: Food items in each source-based phosphorus; Table S2: Association between the total daily phosphorus intake and mortality; Table S3: Association between the proportion of phosphorus intake from plant sources and mortality; Table S4: The association between the proportion of phosphorus intake from animal sources and mortality; Table S5: The association between the proportion of phosphorus intake from other sources and mortality; Item S1: Variables and methods used in the multiple imputation; Item S2: List of clinicians and health care professionals at the participating centres in the DIET-HD Study.
