# Supplementary material for: Dietary Phosphorus, Its Sources, and Mortality in Adults on Haemodialysis: The DIET-HD Study
Source: Nutrients. 2022 Sep 30;14(19):4064. doi: 10.3390/nu14194064 (PMC9572675; doi:10.3390/nu14194064)
Supplement: Supplementary file 1 [file nutrients-14-04064-s001.zip › nutrients-1922166-supplementary.pdf]

Dietary Phosphorus, Its Sources, and Mortality in Adults on Haemodialysis: The DIET-HD Study

Supplementary Materials

Table of Content

Supplementary Figure S1. Flowchart.................................... Error! Bookmark not defined.

Supplementary Table S1. Food items in each source-based phosphorus 3

Supplementary Table S2. Association between total daily phosphorus intake and mortality 5

Supplementary Table S3. Association between proportion of phosphorus intake from plant sources and mortality 7

Supplementary Table S4. Association between proportion of phosphorus intake from animal sources and mortality 9

Supplementary Table S5. Association between proportion of phosphorus intake from other sources and mortality 11

Supplementary Item S1. Variables and methods used in the multiple imputation 13

Supplementary Item S2. List of clinicians and health care professionals at the participating centers in the DIET-HD Study 14

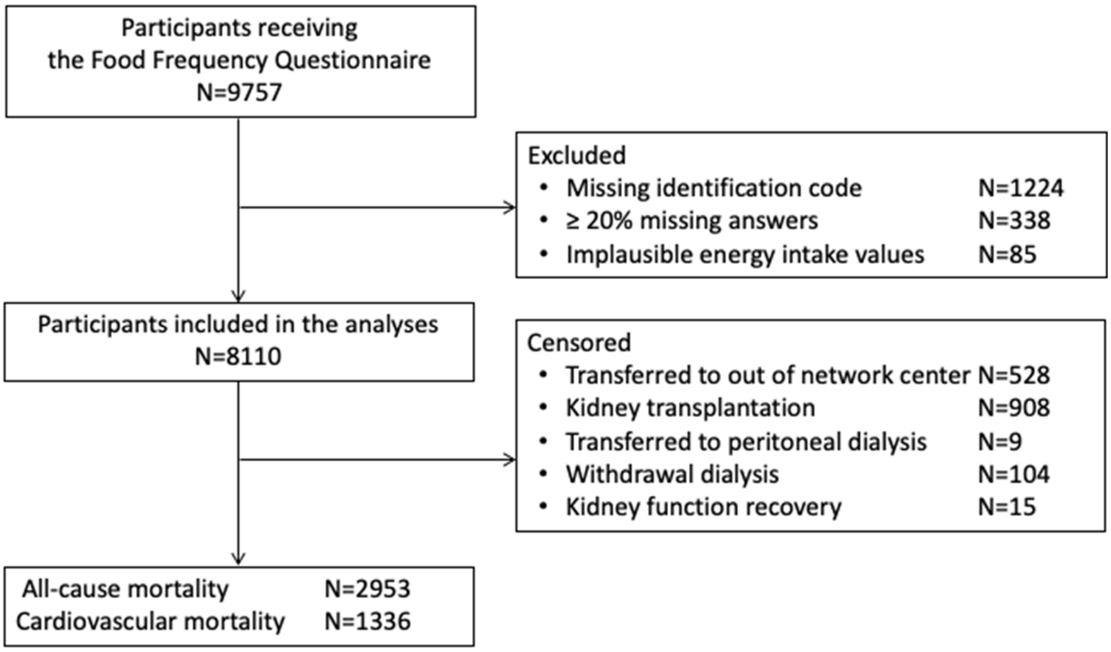

Supplementary Figure S1. Flowchart.

Supplementary Table S1. Food items in each source-based phosphorus.

Phosphorus source	Food items
**Plant-based**	
Bread	Wholemeal or brown bread, white bread, rye bread, naan bread, chapatti, yeast based bread
Cereal	All-bran cereals, cereal bars, wheat germ, Quaker (or other oat cereal) , Cornflakes
Couscous, paster or noodles	Couscous, plain (refined) pasta (e.g., spaghetti), plain wholemeal (unrefined) pasta, filled pasta (with meat/cheese/vegetables), noodles (excluding rice noodles)
Rice	White rice, brown/wholemeal (unrefined) rice, rice noodles
Nuts	Peanuts, cashew nuts, almonds, walnuts
Legumes	Kidney (red), black beans, lentils, chickpeas (also hummus), cluster beans (guar), French beans, fava beans, soya beans
Vegetables	Lettuce, spinach (including lamb's quarters), chard, fenugreek, wild greens (e.g., purslane, watercress), okra, tomato, aubergine, courgette, sweet peppers (e.g., red, green, yellow), cucumber, bitter melon (karela), carrots, parsnip, turnip or swede, artichokes, radish, beetroot, celery, coleslaw, sweet corn, asparagus, herbs (mint, fennel, chive, basil, dill, coriander, parsley), leek, white/other mushrooms, onion, garlic, cauliflower, pumpkin, Brussel sprouts, peas (green), broccoli, cabbage (white, green, red, savoy), stuffed vegetables (vine/green leaves with rice or meat), pickled vegetables (cucumber, radish, cabbage), ginger (in savoury and sweet dishes, in infusion)
Potato	Potato, mashed potatoes, baked/roasted/casserole, chips/French fries, potato in salads, sweet potato, potato tortilla (omelette)
Fruits	Apple, pear, avocado, mango, apricot, nectarine, peach, plum, cherries, rhubarb, berries (e.g. blueberry, strawberry, blackcurrants, blackberry raspberry), banana, melon/ watermelon, grape, squeezed fresh fruit, pineapple, kiwi, lemon, orange, mandarin/tangerine, grapefruit, tinned fruits, raisin, sultana fig, prune olives (e.g. black, green), dates
Soya, tofu	Soy milk, yogurt from soy, tofu
**Animal-based**	
Red meat (beef, veal, lamb, pork, game, etc.)	Hot/cold roast beef, boiled beef, beef steak, fillet, loin beef burger (hamburger) minced, beef meat in stew, casserole, in curry
	Pork cutlet, chop, steak, fillet, loin, pork ribs, mince, meat pies, sausages, veal<br>Small game (e.g., rabbit, goat, pheasant, duck), other game (e.g., deer, moose), lamb (e.g., in stews, kebabs), cured pork (cold or hot-cooked), jamon, ham (e.g., Serrano, prosciutto), dried cured sausages (chorizo, salchichon, salami), Frankfurters, bacon, bacon cubes, smoked lamb

Poultry, chicken	Any poultry with skin, any poultry without skin, chicken (e.g., boiled, roasted, chicken burgers), chicken (e.g., stews or casserole), turkey (e.g., roasted, boiled, strips), any smoked/cured poultry
Liver or offal	Liver (e.g., panita), pates, potted meat, other offal (e.g., tongue, brain, heart, kidney, tripe)
Fish, seafood	Fresh fatty fish (e.g., salmon, tuna, trout, anchovy, herring, mackerel, sardine, Gravlax, eel), fresh white fish (e.g., hake/burbot, cod, haddock, plaice, whiting), other fresh fish/seafood products (e.g., taramasalata), fresh crustaceans and molluscs (e.g., mussel, crab, calamari, octopus, cuttlefish, cured or smoked fatty fish, tinned fish (sardine, tuna, or salmon), tinned crustaceans and molluscs (e.g., mussel, crab, calamari, octopus, cuttlefish, shrimp, clam, sardines, tuna, salmon, kipper), cured or smoked white fish (e.g., cod, Bacalhau)
Eggs	Eggs (fried/poached/boiled/hard boiled/in sandwiches), egg-based savoury dishes, egg-based desserts (e.g., egg cakes, tarts, egg and nuts, sweets)
Milk	Full-fat milk, semi-skimmed milk, skimmed milk, milk fortified with omega 3 fatty acids, yogurt (any type including fromage)
Cheese	Hard cheeses (e.g., Cheddar, Parmesan), soft cheeses (e.g., Brie, Camembert, Philadelphia, Tomini, Boursault, Brinza, Chaource, semi-hard cheeses (e.g., Gouda, Emmental/Edam), cottage cheese (cheese curd) (natural/with scents), fresh cheeses (e.g., Feta, Mozzarella)
Cream	Ice cream, single cream, crème, crème fraiche, sour cream, double or clotted cream
Butter	Low/reduced fat butter, normal butter, lard
**Others**	
Sauce or dressing	Dressing sauces (e.g., French, Caesar, Thousand Island), mayonnaise, white sauce, ketchup, instant soup, pizza (any), Brown sauce
Cakes or pastries	Cakes (e.g., sponge, chocolate,) pastries (e.g., croissants), rolls (with/without stuffing), muffins, donuts, buns (plain or filled), rice pudding, cheese cake, pancakes, plain biscuits (with no fill or cream)
Jam	Jam, marmalade, honey
Sweets	Boiled sweets, toffees, caramels, mixed candies, cereal bars, flapjacks/fruit bar, water ice (ice lolly)
Chocolate	Chocolate snack bars (e.g., Mars bar), dark chocolate, milk chocolate
Margarine or spread	Low-fat margarine, blended spreads, soya-based margarine or spreads, any margarine or vegetable spreads fortified with omega-3
Sweet drinks	Carbonated/soft drinks with sugar, carbonated/soft drinks with artificial sweetener
Tea, beer, wine	Black tea (any), coffee (instant or ground), Greek (Turkish) coffee, green tea, peppermint tea, other herbal infusions, beer (any), red wine, white wine, rose wine, fortified wines (liqueurs (e.g., sherry, port, Madeira)

Supplementary Table S2. Association between total daily phosphorus intake and mortality.

Total daily phosphorus intake (mg/day)	Total	T1 (154-1100; median: 822)	T2 (1101-1747; median: 1388)	T3 (1748-8179; median: 2324)	P for trend	Every SD increase (896 mg/day)	P value
**All-cause mortality**							
Number of observations	8110	2704	2703	2703			
Number of events	2953	943	986	1024			
Event rate per 1000 person-year		108	114	117			
Univariable	8110	1.00 (ref)	1.03 (0.94–1.13)	1.03 (0.94–1.13)	0.49	0.98 (0.94–1.01)	0.23
Multivariable	8110	1.00 (ref)	1.08 (0.98–1.19)	1.22 (1.06–1.40)	0.004	1.16(1.06–1.26)	0.002
Multivariable (adjusted for serum phosphorus additionally)	8110	1.00 (ref)	1.09 (0.98–1.20)	1.24 (1.08–1.42)	0.002	1.17 (1.07–1.28)	0.001
Multivariable (adjusted for protein intake additionally)	8110	1.00 (ref)	1.07 (0.97–1.18)	1.19 (1.03–1.37)	0.02	1.14 (1.02–1.26)	0.02
Competing risk	8110	1.00 (ref)	1.09 (0.99–1.20)	1.26 (1.05–1.41)	0.01	1.17(1.04–1.29)	0.01
**Cardiovascular mortality**							
Number of events	1160	363	355	442			
Event rate per 1000 person-year		42	41	51			
Univariable	8110	1.00 (ref)	1.01 (0.87–1.16)	1.05 (0.90–1.21)	0.54	1.00 (0.95–1.06)	0.91
Multivariable	8110	1.00 (ref)	1.03 (0.88–1.21)	1.15 (0.93–1.42)	0.21	1.18 (1.03–1.36)	0.01
Multivariable (adjusted for serum phosphorus additionally)	8110	1.00 (ref)	1.03 (0.88–1.21)	1.18 (0.95–1.46)	0.14	1.20 (1.05–1.37)	0.009
Multivariable (adjusted for protein intake additionally)	8110	1.00 (ref)	1.03 (0.88–1.21)	1.16 (0.92–1.44)	0.22	1.25 (1.06–1.46)	0.006
Competing risk	8110	1.00 (ref)	1.01 (0.86–1.19)	1.14 (0.90–1.43)	0.32	1.27 (1.09–1.48)	0.002

Multivariable Cox proportional hazard regression analyses fitted using a random effects shared frailty model adjusted for age, sex, physical activity, smoking, body mass index, secondary education, serum albumin, serum haemoglobin, serum calcium, history of cardiovascular disease, diabetes, pulmonary disease, statins, dialysis vintage, vascular access type (fistula versus graft/catheter), Kt/V, energy intake per day, potassium intake gram per day, and Mediterranean diet score. Analysis of cardiovascular mortality was additionally adjusted for alcohol consumption and antihypertensive drugs

Supplementary Table S3. Association between proportion of phosphorus intake from plant sources and mortality.

Proportion of phosphorus intake from plant (percentage)	Total	T1 (0.8–28%; median: 23%)	T2 (29–42%; median: 35%)	T3 (43–99%; 56%)	P for trend	Every 1 SD increase (17%)	P value
**All-cause mortality**							
Number of observations	8110	2704	2703	2703			
Number of events	2953	1034	992	927			
Event rate per 1000 person-year		122	114	103			
Univariable	8110	1.00 (ref)	0.98 (0.89–0.06)	0.92 (0.84–1.01)	0.09	0.97 (0.93–1.01)	0.15
Multivariable	8110	1.00 (ref)	0.96 (0.87–1.05)	0.89 (0.80–0.99)	0.01	0.95 (0.90–0.99)	0.03
Multivariable (adjusted for serum phosphorus additionally)	8110	1.00 (ref)	0.95 (0.87–1.04)	0.88 (0.79–0.99)	0.04	0.94 (0.90–0.98)	0.02
Multivariable (adjusted for protein intake additionally)	8110	1.00 (ref)	0.95 (0.87–1.05)	0.89 (0.79–0.99)	0.04	0.94 (0.89–0.99)	0.02
Competing risk	8110	1.00 (ref)	0.95 (0.87–1.05)	0.88 (0.78–0.99)	0.01	0.93 (0.88–0.98)	0.007
**Cardiovascular mortality**							
Number of events	1160	373	353	434			
Event rate per 1000 person-year		44	41	48			
Univariable	8110	1.00 (ref)	0.92 (0.79–1.06)	0.98 (0.85–1.13)	0.83	1.02 (0.96–1.08)	0.59
Multivariable	8110	1.00 (ref)	0.88 (0.76–1.03)	0.94 (0.79–1.11)	0.46	0.99 (0.92–1.06)	0.78
Multivariable (adjusted for serum phosphorus additionally)	8110	1.00 (ref)	0.88 (0.75–1.03)	0.93 (0.79–1.10)	0.42	0.98 (0.92–1.05)	0.71
Multivariable (adjusted for protein intake additionally)	8110	1.00 (ref)	0.88 (0.76–1.03)	0.86 (0.72–1.03)	0.09	0.93 (0.85–1.00)	0.07

Competing risk	8,110	1.00 (ref)	0.89 (0.76–1.03)	0.92 (0.78–1.09)	0.41	0.96 (0.91–1.06)	0.69

Multivariable Cox proportional hazard regression analyses fitted using a random effects shared frailty model adjusted for age, sex, physical activity, smoking, body mass index, secondary education, history of cardiovascular disease, hypertension, diabetes, pulmonary disease, cancer, serum haemoglobin, serum albumin, serum calcium, statins, phosphate-binders (any or none), vascular access type (fistula versus graft/catheter), dialysis vintage, Kt/V, total energy intake per day and potassium intake gram per day, and Mediterranean diet score (we included total daily phosphorus intake as covariate when investigating the association between proportion of phosphorus from specific sources and mortality). Analysis of cardiovascular mortality was additionally adjusted for alcohol consumption and antihypertensive drugs.

Supplementary Table S4. Association between proportion of phosphorus intake from animal sources and mortality.

Proportion of phosphorus intake from animal source (percentage)	Total	T1 (0–45%; median: 34%)	T2 (46–61%; median: 55%)	T3 (62–99%; median: 70%)	P for trend	Every 1 SD increase (17%)	P value
**All-cause mortality**							
Number of observations	8110	2704	2703	2703			
Number of events	2953	981	972	1000			
Event rate per 1000 person-year		110	113	117			
Univariable random effect	8110	1.00 (ref)	1.03 (0.94–1.12)	1.06 (0.97–1.17)	0.21	1.03 (1.00–1.07)	0.08
Multivariable random effect	8110	1.00 (ref)	1.01 (0.91–1.12)	0.96 (0.85–1.09)	0.54	1.01 (0.96–1.05)	0.46
Multivariable random effect (adjusted for serum phosphorus)	8110	1.00 (ref)	1.02 (0.91–1.12)	1.00 (0.88–1.13)	0.61	1.02 (0.97–1.07)	0.45
Multivariable (adjusted for protein intake additionally)	8110	1.00 (ref)	1.01 (0.91–1.11)	0.96 (0.85–1.09)	0.53	1.02 (0.96–1.07)	0.58
Competing risk	8110	1.00 (ref)	1.03 (0.90–1.13)	1.01 (0.89–1.14)	0.51	1.01 (0.95–1.06)	0.46
**Cardiovascular mortality**							
Number of events	1160	459	366	335			
Event rate per 1000 person-year		51	42	39			
Univariable random effect	8110	1.00 (ref)	0.96 (0.83–1.10)	0.99 (0.85–1.15)	0.87	1.01 (0.94–1.07)	0.86
Multivariable random effect	8110	1.00 (ref)	0.94 (0.81–1.09)	0.92 (0.78–1.09)	0.35	0.99 (0.93–1.06)	0.79
Multivariable random effect (adjusted for serum phosphorus)	8110	1.00 (ref)	0.94 (0.81–1.09)	0.93 (0.78–1.10)	0.37	0.99 (0.93–1.06)	0.83
Multivariable (adjusted for protein intake additionally)	8110	1 (Ref)	1.02 (0.86–1.20)	1.00 (0.82–1.21)	0.99	1.07 (0.97–1.16)	0.17

Competing risk	8110	1.00 (ref)	0.93 (0.80–1.08)	0.94 (0.78–1.11)	0.39	1.00 (0.94–1.07)	0.85

Multivariable Cox proportional hazard regression analyses fitted using a random effects shared frailty model adjusted for age, sex, physical activity, smoking, body mass index, secondary education, history of cardiovascular disease, hypertension, diabetes, pulmonary disease, cancer, serum haemoglobin, serum albumin, serum calcium, statins, phosphate-binders (any or none), vascular access type (fistula versus graft/catheter), dialysis vintage, Kt/V, total energy intake per day and potassium intake gram per day, and Mediterranean diet score (we included total daily phosphorus intake as covariate when investigating the association between proportion of phosphorus from specific sources and mortality). Analysis of cardiovascular mortality was additionally adjusted for alcohol consumption and antihypertensive drugs.

Supplementary Table S5. Association between the proportion of phosphorus intake from other sources and mortality.

Proportion of phosphorus intake from other source (percentage)	Total	T1 (0–3%; median: 2%)	T2 (4–8%; median: 6%)	T3 (9–88%; median: 14%)	P for trend	Every 1 SD increase (9%)	P value
**All-cause mortality**							
Number of observations	8110	2704	2703	2703			
Number of events	2953	994	941	1018			
Event rate per 1000 person-year		115	106	118			
Univariable	8110	1.00 (ref)	0.83 (0.76–0.90)	0.86 (0.78–0.94)	<0.01	0.99 (0.95–1.02)	0.58
Multivariable	8110	1.00 (ref)	0.99 (0.90–1.08)	1.12 (1.01–1.24)	0.03	1.06 (1.02–1.10)	0.006
Multivariable (adjusted for serum phosphorus additionally)	8110	1.00 (ref)	0.99 (0.90–1.08)	1.12 (1.01–1.25)	0.004	1.06 (1.02–1.10)	0.004
Multivariable (adjusted for protein intake additionally)	8110	1.00 (ref)	0.99 (0.89–1.08)	1.13 (1.02–1.25)	0.03	1.06 (1.02–1.10)	0.003
Competing risk	8110	1.00 (ref)	0.99 (0.91–1.08)	1.11 (1.01–1.23)	0.003	1.07 (1.03–1.11)	0.005
**Cardiovascular mortality**							
Number of events	1160	380	366	414			
Event rate per 1000 person-year		44	41	48			
Univariable	8110	1.00 (ref)	0.81 (0.70–0.94)	0.81 (0.69–0.94)	<0.01	0.96 (0.90–1.01)	0.17
Multivariable	8110	1.00 (ref)	0.96 (0.83–1.11)	1.05 (0.90–1.24)	0.52	1.03 (0.96–1.09)	0.35
Multivariable (adjusted for serum phosphorus additionally)	8110	1.00 (ref)	0.96 (0.83–1.12)	1.06 (0.90–1.25)	0.48	1.04 (0.97–1.10)	0.29
Multivariable (adjusted for protein intake additionally)	8110	1.00 (ref)	0.98 (0.85–1.15)	1.08 (0.92–1.28)	0.35	1.03 (0.96-1.09)	0.45
Competing risk	8110	1.00 (ref)	1.00 (0.86–1.16)	1.09 (0.92–1.30)	0.31	1.04 (0.95-1.11)	0.38

Multivariable Cox proportional hazard regression analyses fitted using a random effects shared frailty model adjusted for age, sex, physical activity, smoking, body mass index, secondary education, history of cardiovascular disease, hypertension, diabetes, pulmonary disease, cancer, serum haemoglobin, serum

albumin, serum calcium, statins, phosphate-binders (any or none), vascular access type (fistula versus graft/catheter), dialysis vintage, Kt/V, total energy intake per day and potassium intake gram per day, and Mediterranean diet score (we included total daily phosphorus intake as covariate when investigating the association between proportion of phosphorus from specific sources and mortality). Analysis of cardiovascular mortality was additionally adjusted for alcohol consumption and antihypertensive drugs.

Supplementary Item S1. Variables and methods used in the multiple imputation.

In this subset population, most covariates had fewer than 5% missing data, except for serum albumin (24%), educational level (25%), smoking (23%), physical activity (24%), and statin (23%). Data were assumed to be missing at random and 30 datasets were input using multiple imputation chained equation. We used linear regression for the input of continuous variables as no covariates with missing data had a skewed distribution. For categorical variables, the input was conducted by logistic regression (binary) or ordinal logistic regression (ordinal categorical with more than two levels). All covariates with missing values used in the multivariable analysis were included in the imputation models. Daily phosphorus intake, age, sex, dialysis vintage, comorbidities (history of cardiovascular disease, history of cardiovascular disease, hypertension, diabetes, pulmonary disease, gastrointestinal disease, cancer, liver disease), antihypertensive medication, statin, phosphate binders(any or none), total energy intake (1000 kcal/day), weight change in 3 months prior, normalised protein catabolic rate, country, time at risk of death, and Nelson–Aalen estimator for outcome of interest (all-cause mortality or cardiovascular mortality) were included as auxiliary variables.

Supplementary Item S2. List of clinicians and health care professionals at the participating centres in the DIET-HD Study.

Argentina: R Gelfman, E Celia, A Badino, L Petracci, C Villareal, M Soto, M Arias, F Vera, V Quispe, S Morales, D Bueno, R Bargna, G Peñaloza, L Alcalde, J Dayer, A Milán, N Centurión, A Ramos, E De Orta, S Menardi, N Austa Bel, E Marileo, N Junqueras, C Favalli, R Trioni, G Valle, M López, C Marinaro, A Fernandez, J Corral, E Nattiello, S Marone, J García, G Carrizo, P González, O Delicia, M Maza, M Chauque, J Mora, D Grbavac, L López, M Alonso, C Villalba, M Simon, M Cernadas, C Moscatelli, I Vilamajó, C Tursky, M Martínez, F Villalba, D Pereira, S Araujo, H López, V Alonso, B Vázquez, M Rapetti, S Raña, M Capdevila, C Ljubich, M Acosta, M Coombes, V Doria, M Ávila, D Cáceres, E Geandet, C Romero, E Morales, C Recalde, S Marone, M Casanú, B Lococo, O Da Cruz, C Focsaner, D Galarce, L Albarracín, E Vescovo, M Gravielle, D Florio, L Baumgart, M Corbalán, V Aguilera, O Hermida, C Galli, L Ziombra, A Gutierrez, S Frydelund, A Hardaman, A Maciel, M Arrigo, C Mato Mira, J Leibovich, R Paparone, E Muller, A Malimar, I Leocadio, W Cruz, S Tirado, A Peñalba, R Cejas, S Mansilla, C Campos, E Abrego, P Chávez, G Corpacci, A Echavarría, C Engler, P Vergara, M Hubeli, G Redondo, B Noroña;

France: P Stroumza, C Boriceanu, M Lankester, JL Poignet, Y Saingra, M Indreies, J Santini, Mahi A, A Robert, P Bouvier, T Merzouk, F Villemain, A Pajot, F Tollis, M Brahim-Bounab, A Benmoussa, S Albitar, MC Guimont, P Ciobotaru, A Guerin, M Diaconita; Germany: M Hansis, SH Hoischen, J Saupe, I Ullmann S Grosser, J Kunow, S Grueger, D Bischoff, J Benders, P Worch, T Pfab, N Kamin, M Roesch, M May;

Hungary: M Török, K Albert, I Csaszar, E Kiss, D Kosa, A Orosz, J Redl, L Kovacs, E Varga, M Szabo, K Magyar, E Zajko, A Bereczki, J Csikos, E Kerekes, A Mike, K Steiner, E Nemeth, K Tolnai, A Toth, J Vinczene, Sz Szummer, E Tanyi, M Szilvia;

Italy: L Gargano, AM Murgo, N Sanfilippo, N Dambrosio, C Saturno, G Matera, M Benevento, V Greco, G di Leo, S Papagni, F Alicino, A Marangelli, F Pedone, AV Cagnazzo, R Antinoro, ML Sambati, C Donatelli, F Ranieri, F Torsello, P Steri, C Riccardi, A Flammini, L Moscardelli, E Boccia, M Mantuano, R Di Toro Mammarella, M Meconizzi, R Fichera, A D'Angelo, G Latassa, A Molino, M Fici, A Lupo, G Montalto, S Messina, C Capostagno, G Randazzo, S Pagano, G

Marino, D Rallo, A Maniscalco, OM Trovato, C Strano, A Failla, A Bua, S Campo, P Nasisi, A Salerno, S Laudani, F Grippaldi, D Bertino, DV Di Benedetto, A Puglisi, S Chiarenza, M Lentini Deuscit, CM Incardona, G Scuto, C Todaro, A Dino, D Novello, A Coco;

Poland: AB Skublewsk, J Duława, E Bocheńska-Nowacka, A Jaroszyński, J Drabik, M Wypych-Birecka, D Daniewska, M Drobisz, K Doskocz, G Wyrwicz-Zielińska, A Kosicki, W Ślizień, P Rutkowski, S Arentowicz, S Dzimira, M Grabowska, J Ostrowski, A Całka, T Grzegorczyk, W Dżugan, M Mazur, M Myślicki, M Piechowska, D Kozicka;

Portugal: V de Sá Martins, L Aguiar, AR Mira, B Velez, T Pinheiro;

Romania: D Timofte, E Agapi, CL Ardelean, A Baidog, G Bako, M Barb, A Blaga, E Bodurian, V Bumbea, E Dragan, D Dumitrache, L Florescu, N Havasi, S Hint, R Ilies, AGM Mandita, RI Marian, SL Medrihan, L Mitea, S Mitea, R Mocanu, DC Moro, M Nitu, ML Popa, M Popa, E Railean, AR Scuturdean, K Szentendrey, CL Teodoru, A Varga;

Spain: AG Bernat, D Del Castillo, M García, M Olaya, V Abujder, J Carreras, A López, F Ros, G Cuesta, A García, E Orero, E Ros, S Bea, JL Pizarro, S Luengo, A Romero, M Navarro, L Cermeño, A Rodriguez, D Lopez, A Barrera, F Montoya, J Tajahuerce, M Carro, MQ Cunill, S Narci, T Ballester, MJ Soler, S Traver, PP Buta, L Cucuiat, L Rosu, I Garcia, CM Gavra, R Gonzalez, S Filimon, M Peñalver, V Benages, MI Cardo, E García, P Soler, E Fernnandez, F Popescu, R Munteanu, E Tanase, F Sagau, D Prades, S Esteller, E Gonzalez, R Martinez, A Diago, J Torres, E Perez, C Garcia, I Lluch, J Forcano, M Fóns, A Rodríguez, NA Millán, J Fernández, B Ferreiro, M Otero, V Pesqueira, S Abal, R Álvarez, C Jorge, I Rico, J de Dios Ramiro, L Duzy, A Soto, JL Lopez, Y Diaz, I Herrero, M Farré, C Blasco, S Ferrás, MJ Agost, C Miracle, J Farto;

Sweden: E Fabricius, J Goch, KS Katzarski, A Wulcan, C Wollheim, J Hegbrant;

Turkey: T Ecder, H Akbiber, H Arslan, L Bicen, A Buyukkiraz, R Celik, I˙S Dogan, S Erkalkan, A Ertas, U Hark, E Iravul, M Karakaya, K Mengu, S Ongun, Z Ozkan, A Ozlu, N Ozveren, HM Sifil, N Sonmez Turksoz, Z Yilmaz.
